# Detection of *Chlamydia psittaci* in both blood and bronchoalveolar lavage fluid using metagenomic next-generation sequencing: A case report

**DOI:** 10.1097/MD.0000000000026514

**Published:** 2021-07-09

**Authors:** Yingpu Yuan, Xiaobo Zhang, Chunmei Gui

**Affiliations:** a Department of Critical Care Medicine; b Department of Neurology, The First People's Hospital of Changde City, Changde, Hunan, China.

**Keywords:** blood, bronchoalveolar lavage fluid, *Chlamydia psittaci*, metagenomic next-generation sequencing, sepsis

## Abstract

**Rationale:**

*Chlamydia psittaci* (*C psittaci*) is a gram-negative obligate intracellular parasite, with birds as main hosts. The main route of infection in humans is inhalation of aerosols from contaminated animal excreta through the respiratory tract. The main manifestation of *C psittaci* infection is pneumonia. Patients suffering from severe infection are prone to sepsis and multiple organ failure. We report a case of simultaneous detection of *C psittaci* in blood and bronchoalveolar lavage fluid using metagenomic next-generation sequencing (mNGS) technology.

**Patient concerns:**

The 71-year-old male patient was a farmer with a long history of raising poultry and initial symptoms of fever and muscle pain accompanied by limb weakness and paroxysmal cough.

**Diagnoses:**

The patient was diagnosed with sepsis, severe pneumonia, and multiple organ failure.

**Interventions:**

Anti-infective therapy with doxycycline and meropenem was applied.

**Outcomes:**

The patient's body temperature and infection indicators improved and the chest X-ray findings showed the amelioration of lesions after 18 days of treatment. The patient was discharged without treatment on hospital day 19 due to financial constraints and subsequently died after 7 days.

**Lessons:**

mNGS is an excellent diagnostic tool when specific pathogens are undetected by traditional assays.

## 1 Introduction

*Chlamydia psittaci* (*C psittaci*) is a zoonotic pathogen with birds as primary hosts. The main route of infection in humans is inhalation of aerosols from contaminated animal excreta through the respiratory tract.^[1]^*C psittaci* may cause severe diseases in people who have come into contact with birds. The *C psittaci* infection in humans may lead to severe conditions and complications, including sepsis and multiple organ failure. This parasite is more pathogenic and reproduces at a faster rate than other chlamydial species and therefore causes more severe inflammatory response that can lead to higher mortality.^[2]^ However, the difficulty in identifying psittacosis with traditional detection methods usually leads to misdiagnosis of this condition in clinical practice. Therefore, a method for rapid detection of *C psittaci* is necessary. Metagenomic next-generation sequencing (mNGS) is a new tool that can rapidly and accurately identify potential pathogens, including viruses, bacteria, fungi, and parasites and mycoplasma.^[3]^ The precision of mNGS is enhanced because it relies on information in DNA sequences of patient tissues and body fluids.^[4]^

Gu et al^[5]^ successfully identified the presence of *C psittaci* from biopsied lung tissue and bronchoalveolar lavage fluid using mNGS. We report a case of *C psittaci* pneumonia dignosed via mNGS analysis of both blood and bronchoalveolar lavage fluid samples in this study. To the best of our knowledge, reports on the simultaneous detection of pathogens using mNGS in the blood and bronchoalveolar lavage fluid of *C psittaci*-infected patients are limited. This study was approved by the Ethics Committee of the First People's Hospital of Changde, and the disclosure of clinical data was recognized by patients’ families.

## 2 Case presentation

We report the case of a 71-year-old man diagnosed with *C psittaci* pneumonia with complications of sepsis and multiple organ failure.

**Figure 1:**
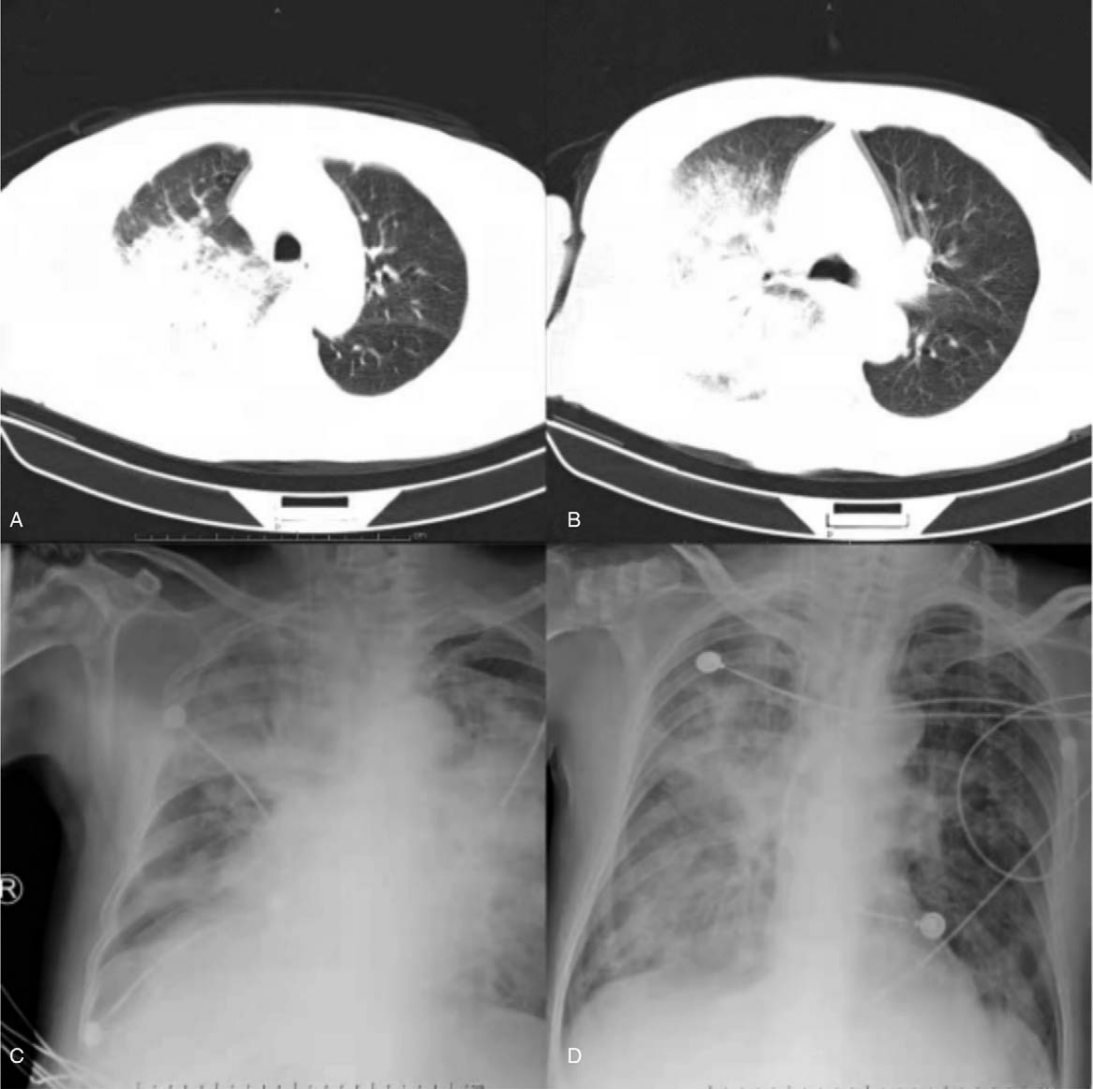
Chest CT and X-ray of the patient: A and B, On the day of admission, C, 4 days after doxycycline treatment, and D, 7 days after doxycycline treatment, suggesting that the lung infection improved.

**Figure 2:**
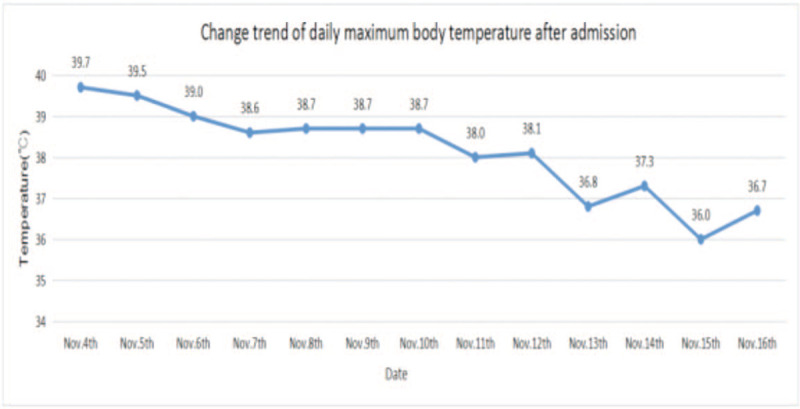
After taking doxycycline for 3 days (Nov. 11), the maximum daily body temperature of this patient was significantly lower than before, indicating that the treatment was effective.

**Figure 3:**
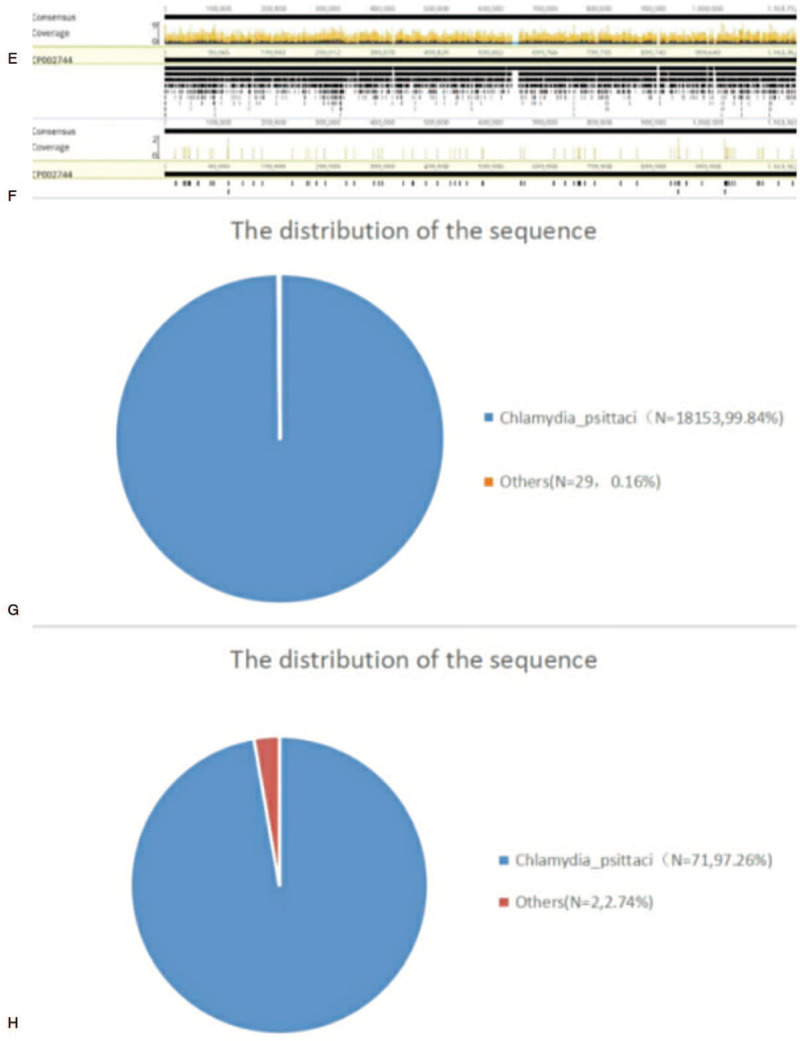
Diagnosis of *Chlamydia psittaci* in blood and bronchoalveolar lavage fluid using next generation sequencing. (E, G) A total of 18,153 sequence reads corresponded to *C psittacil*, accounting for 99.84% of microbial reads by NGS in the bronchoalveolar lavage fluid. (F, H) A total of 71 sequence reads corresponded to *C psittacil*, accounting for 97.26% of microbial reads by NGS in the blood sample. NGS = next-generation sequencing.

The patient suffering from gout was a farmer with a long history of raising poultry and without other known medical conditions. The patient visited a local hospital for treatment on October 29, 2020 due to fever with a temperature of nearly 39 °C accompanied by muscle soreness and paroxysmal cough. The condition of the patient deteriorated gradually after 5 days and was transferred to the emergency department of our hospital for further treatment. The lung computed tomography (CT) scan showed pulmonary infection (Fig. 1). Blood routine analysis presented a white blood cell (WBC) count of 7.84 × 10^9^/L and N% of 91.9%. The patient demonstrated decreased blood oxygen index, needed ventilator support, and was sent to the intensive care unit in the emergency department. We completed the examination of bronchoalveolar lavage fluid, sputum, and blood culture after admission. At this time, the patient was treated with meropenem. The patient still exhibited lasting high fever in the next 2 days (Fig. 2). Serum levels of inflammatory makers increased gradually (Table 1). However, pathogens were absent in repeated sputum, bronchoalveolar lavage fluid, and blood cultures of the patient. We subsequently performed mNGS of bronchoalveolar lavage fluid and blood samples. mNGS of blood and bronchoalveolar lavage fluid revealed *C psittaci* on the fourth day after admission. A total of 18,153 sequence reads corresponding to *C psittaci* accounted for 99.84% of microbial reads via mNGS in the bronchoalveolar lavage fluid. Seventy-one sequence reads corresponding to *C psittaci* accounted for 97.26% of microbial reads via mNGS in the blood sample (Fig. 3). Combined with the medical history, we concluded that the patient was infected by *C psittaci*. Doxycycline combined with meropenem was then administered. Temperature and infection indicators decreased on the tenth day after admission. The chest X-ray results showed that lesions in both lungs are improving, thereby indicating the effectiveness of the treatment (Fig. 1). However, the patient was discharged without treatment on hospital day 19 due to financial constraints and subsequently died after 7 days.

**Table 1:** The patient's white blood cell (WBC) count was normal during hospitalization.

Inflammatory makers	Nov.4	Nov.7	Nov.9	Nov.12	Nov.14	Nov.16
WBC (10^9^/L)	6.43	6.04	5.30	9.25	8.75	3.04
N (%)	92.80	94.30	91.80	96.11	95.40	87.90
CRP, mg/L	385.21	520.43	449.72	473.63	198.56	70.64
PCT, ng/mL	24.55	58.33	24.05	28.64	12.86	6.52

The proportion of neutrophils (N), C-reactive protein (CRP), and procalcitonin (PCT) were higher in the first few days of hospitalization. After the use of doxycycline, the serum levels of inflammatory makers of the patients decreased significantly, indicating that the anti-infective treatment was effective. CRP = c-reactive protein, N = neutrophils, PCT = procalcitonin, WBC = white blood cell.

Pathogens were undetected in sputum, bronchoalveolar lavage fluid, and blood culture during hospitalization. Meanwhile, the confirmed presence of *C psittaci* in the blood and bronchoalveolar lavage fluid through mNGS analysis indicated that mNGS is more sensitive than conventional detection.

## 3 Discussion and conclusion

The patient in this case report presented fever and muscle aches accompanied by limb weakness and paroxysmal cough as first symptoms, followed by sepsis and multiple organ failure that required ventilator support. The patient's lung CT findings revealed pneumonia on the day of admission. Routine blood testing showed that the white blood cell count was normal with a proportion of neutrophils of 85.6%. However, the significantly high C-reactive protein and procalcitonin indicated a severe case of pneumonia. The patient was initially treated with meropenem. Alveolar lavage fluid, sputum, and blood cultures were all negative during hospitalization. mNGS of blood and bronchoalveolar lavage fluid was performed for rapid and accurate diagnosis. The mNGS results of both blood and bronchoalveolar lavage fluid 4 days after admission confirmed the presence of *C psittaci*. Tetracyclines, macrolides, and quinolones are effective drugs against *C psittaci*.^[6]^ Hence, we used doxycycline combined with meropenem for anti-infective therapy. The patient's symptoms and laboratory and chest radiograph findings improved significantly 10 days after admission. However, the patient's family cannot afford high medical costs and gave up treatment.

*C psittaci* can be divided into 10 genotypes with different preferences for host species.^[7]^ Genotypes A and E can infect humans. Humans can be infected with *C psittaci* or commonly known as psittacosis. *C psittaci* is transmitted to the reticuloendothelial system by contaminated aerosols. The lung is the typical site of *C psittaci* infection. *C psittaci* pneumonia accounts for approximately 1% of community-acquired pneumonia.^[8,9]^ Clinical manifestations usually include fever, intense headache, myalgia, and dry cough.^[10,11]^ Methods for diagnosing *C psittaci* infections have attracted considerable research attention for years due to the absence of an ideal tool until recently. Although conventional diagnostic methods mainly include culture, serological detection, and PCR-based methods, they easily produce false negative results.^[5]^ Pendleton et al^[2]^ reported that *C psittaci* is more pathogenic than other C. species. Moreover, *C psittaci* predisposes to severe inflammatory response and causes high mortality. Therefore, detecting the pathogen as early as possible and achieving accurate diagnosis are essential in providing targeted treatment.

Although standard microbiological diagnostic techniques are generally unable to detect particular pathogens,^[2]^ the successful identification of causative pathogens using mNGS in such cases indicates that mNGS an excellent diagnostic tool.^[12]^ The parallel sequencing of nucleic acids (DNA and/or RNA) from all samples in the detection of pathogenic microorganisms via mNGS can accurately diagnose pathogens.^[12]^ The identification of nearly 89% of infected patients through mNGS is higher than that via traditional pathogen detection at 25.73% (*P* < .001).^[13]^ This finding reflected the significantly better sensitivity of mNGS for detecting pathogens than that of traditional pathogen detection. However, mNGS is limited by the requirement of sequencing of the human host background and high cost.^[4]^ Gu et al^[5]^ successfully identified the presence of *C psittaci* from the biopsy of lung tissue and bronchoalveolar lavage fluid using mNGS. A critical review of the literature showed that studies on the use of mNGS-based simultaneous confirmation of pathogen presence in blood and bronchoalveolar lavage fluid are limited. A case of severe pneumonia complicated by sepsis and multiple organ dysfunction due to *C psittaci* was diagnosed in our department through mNGS of blood and bronchoalveolar lavage fluid.

This study suggested that mNGS is a new test method for accurate clinical diagnosis of pathogens, even in rare pathogen infections.^[14,15]^ mNGS can provide high detection rate and help initiate treatment early because it can simultaneously examine nucleic acid sequences of multiple pathogenic microorganisms. Hence, patient outcomes can improve and the use of nontargeted antibiotics can be minimized.

## Acknowledgment

The authors would like to thank the patient for their participation in this study.

## Author contributions

**Data curation:** Xiaobo Zhang, Chunmei Gui.

**Writing – original draft:** Yingpu Yuan, Xiaobo Zhang.
